# DNA-Directed Assembly of Carbon Nanotube–Protein Hybrids

**DOI:** 10.3390/biom11070955

**Published:** 2021-06-29

**Authors:** Mark Freeley, Rebecca E. A. Gwyther, D. Dafydd Jones, Matteo Palma

**Affiliations:** 1 Department of Chemistry, Queen Mary University of London, London E1 4NS, UK; m.freeley@qmul.ac.uk; 2 Molecular Biosciences Division, School of Biosciences, Cardiff University, Cardiff CF10 3AX, UK; GwytherRE@cardiff.ac.uk

**Keywords:** single-walled carbon nanotube, protein, DNA, hybrid, SWCNT, GFP

## Abstract

Here, we report the controlled assembly of SWCNT–GFP hybrids employing DNA as a linker. Two distinct, enriched SWCNTs chiralities, (6,5), (7,6), and an unsorted SWCNT solution, were selectively functionalized with DNA and hybridized to a complementary GFP^DNA^ conjugate. Atomic force microscopy images confirmed that GFP attachment occurred predominantly at the terminal ends of the nanotubes, as designed. The electronic coupling of the proteins to the nanotubes was confirmed via in-solution fluorescence spectroscopy, that revealed an increase in the emission intensity of GFP when linked to the CNTs.

## 1. Introduction

The optical and electronic properties of single-walled carbon nanotubes (SWCNTs) have long been the focus of nanomaterials research, including the design of biosensors [1,2,3,4,5]. Consequently, modifying SWCNTs with biorecognition elements or biomolecules themselves is a key step in this process [6,7,8,9,10,11], and the functionalization of SWCNTs has been tailored through both covalent and non-covalent approaches [12,13,14,15,16,17,18,19,20].

In recent years, different studies have demonstrated the importance of controlling the position and orientation of the biomolecule relative to the SWCNT: biomolecules, and particularly proteins, are not uniform structures, as nanoparticles are, and therefore the point at which they are tethered is important to consider [7,11,21]. This has been shown to have significant effects in the protein/SWCNT interactions. Zubkovs et al. and Thomas et al. [11,21] have shown how the site-specific attachment of a fluorescent protein affects the optical behavior of both protein and SWCNTs; this was demonstrated by investigating the change in a fluorescent protein function and, in the case of Zubkovs et al., of (7,6) SWCNTs within a mixture of nanotubes. There are other examples of changes in the fluorescent behavior of SWCNTs due to biofunctionalization, but these investigations are typically carried out with unsorted SWCNTs; hence, the interaction can only be considered an average response to each species in the mixture. 

In this regard, there is an interest in tailoring the chirality of SWCNTs for different applications. In a broader sense, separating metallic and semiconducting SWCNTs is desirable because of different electronic applications. From an optical perspective, SWCNTs with semiconducting behavior are particularly interesting, where their chirality determines the bandgap of the nanotube and hence its emission and absorbance spectra. This has led to their use as optical sensors and markers for bioimaging, where their emission in the IR can be beneficial due to higher tissue penetration [22,23,24].

Significant progress has been made in the purification of SWCNTs into individual chiralities, where aqueous two-phase polymer (ATP) separations have been shown to be an effective approach [25,26,27,28,29,30]. Among the successful approaches, the DNA-wrapping of SWCNTs has been shown to be efficient, where the DNA wraps around each chirality of the SWCNT with varying preference [31]. SWCNTs can then be driven between aqueous polymer phases with the use of modulating agents, as a result of the stability of each SWCNT/DNA complex. Using this system, single chirality SWCNTs can be isolated with high purity in water, making them biocompatible.

Here, we sought to control the assembly of green fluorescent protein (GFP) [32,33] predominantly to the terminal end of individual SWCNTs, employing DNA as an addressable linker molecule. The superfolding variant of GFP [34] was bioengineered to contain an azide functional group at a specific residue to allow a bio-orthogonal “1 + 1” click chemistry reaction with cyclooctyne-modified oligonucleotide (oligo) [35,36,37]. A complementary azide-modified oligo was tethered to the terminal end of an SWCNT via a UV-induced cycloaddition reaction [9]. End-functionalization of SWCNTs was achieved as the DNA-wrapping of SWCNTs passivated the sidewalls, while the defects on the ends of the tubes remain exposed [7,8,9]. The bioorganic hybrids were then assembled via DNA hybridization of the complementary strands. We employed enriched (6,5), (7,6), and unsorted SG65i (containing (6,5), (7,5), and (7,6) species) SWCNTs and demonstrated their coupling with GFP. We observed an increase in emission intensity for all the GFP–CNT nanohybrids, suggesting electronic coupling between the protein and all the SWCNT chiralities employed.

## 2. Materials and Methods

### 2.1. Purification of SWCNTs by Chirality

Single-walled carbon nanotubes (SWCNTs; 1 mg/mL) were wrapped with DNA (2.5 mg/mL, Integrated DNA Technologies, Leuven, Belgium; 30 mM NaCl) via bath sonication (sonic bath model) for 90 min. The solution of dispersed nanotubes was centrifuged (centrifuge model) at 13,000 rpm for 60 min. The supernatant which contained dispersed SWCNTs was carefully pipetted into a new microcentrifuge tube and the pellet of aggregated SWCNTs was discarded.

SWCNTs were purified using a method adapted from Lyu et al. [25] Specifically, (6,5) SWCNTs were purified from SG65i SWCNTs (Sigma Aldrich, Gillingham, UK) wrapped with (TCTTTT)_2_TCT. The (7,6)-enriched SWCNTs were obtained from SG76 SWCNTs (Sigma Aldrich) wrapped with (ATT)_4_-NH_2_. Then (7:3) and (10:0) solutions were made up using Dextran (250 kDa) and PEG (1.5 kDa). Dispersed SWCNTs (45 µL) were mixed with vortexed (7:3) solution (140 µL) and an aqueous solution of 10% polyvinyl pyrrolidone (PVP; 0.33 µL; 40 kDa), after which the combined solutions were vortexed for 30 s and centrifuged for 2 min at 13,000 rpm to separate the solution into two phases. The top phase was removed to a new tube and the volume removed was replaced with blank top phase of the (10:0) solution. This was repeated until no SWCNTs remained in the bottom phase. Extracted SWCNTs were characterized by UV–Vis spectroscopy (Shimadzu UV-3600i Plus, Milton Keynes, UK). To remove polymers, sodium thiocyanate (NaSCN) was added to the isolated fractions to assist in precipitating the SWCNTs with centrifugation. The pellet of SWCNTs was washed with solutions of NaSCN (3M) and water, being careful not to agitate the pellet. After several washes, the pellet was redispersed using the dispersing solution of DNA and NaCl with sonication if necessary. Pure SWCNTs were further purified against water using dialysis membranes (Thermo Scientific™ Slide-A-Lyzer™ MINI Dialysis Devices, 20 kDa MWCO, 0.1 mL, Fisher Scientific, Loughborough, UK).

### 2.2. Functionalization of GFP with DNA

(1R,8S,9s)-Bicyclo[6.1.0]non-4-yn-9-ylmethyl N-succinimidyl carbonate (BCN-NHS ester; 0.25 mg, Sigma Aldrich, Gillingham, UK) was dissolved in DMSO (14 µL) and was subsequently added to sodium tetraborate buffer (75 µL; 0.1 M; pH 8.5). Water (7 µL) and amine-DNA (4 µL; 25 µg/µL; oligo (1), see Table 1) were then added, and the solution was shaken at room temperature overnight. The BCN-modified DNA was precipitated by adding aqueous NaCl (10 µL; 3 M) and ethanol (275 µL) and centrifuging at 13,000 rpm for 30 min. The supernatant was removed and discarded, and the pellet was washed with cold 70% ethanol. The pellet was then resuspended in water and filtered three times with a microcentrifuge filter (Millipore Amicon Ultra 0.5 3 kDa, Merck, Watford, UK).

Functionalization of GFP with BCN-labelled oligonucleotides was performed essentially as described previously [37]. The superfolding version of GFP with AzF at residue 204 (GFP^204AzF^) was produced as described previously [35,36]. GFP^204AzF^ was mixed with BCN–DNA in a 1:10 ratio in Dulbecco’s phosphate-buffered saline (DPBS) and shaken overnight at room temperature. The GFP–DNA conjugate (GFP^DNA^) was then purified by native (non-denaturing) polyacrylamide gel electrophoresis (PAGE; Biorad Mini-PROTEAN) using a 10% gel. Bands corresponding to GFP^DNA^ were excised from the gel and the conjugate was extracted using the crush and soak method in DPBS. GFP^DNA^ in solution was separated from the crushed gel using a Corning centrifuge filter (0.1 µm pore size), and the conjugate was concentrated using a 10 kDa centrifuge filer (Millipore amicon ultra 0.5 10 kDa). UV–Vis was used to quantify the concentration of GFP^DNA^ using an extinction coefficient of 51,000 at 485 nm [36,37].

### 2.3. Functionalization of DNA-Wrapped SWCNTs with Azide-Modified DNA

SWCNTs were functionalized with azide-modified DNA (oligo (2), see Table 1) by adapting a previously used method [9]. SWCNTs (~5 µg/mL) were mixed with azide-modified DNA (250 nM final concentration) and 10x DPBS (2.5 µL) and made up to a final volume of 25 µL with MilliQ water. Three different SWCNT samples were used for this: enriched (6,5), enriched (7,6), and unsorted SG65i SWCNTs [1]. The concentration of each variant was normalized according to the E_11_ absorbance peak. Considering that SG65i SWCNTs comprise approximately 40% (6,5) chirality, for the unsorted samples the SWCNTs were diluted to have a E_11_ absorbance of 0.4 times that of the chirally enriched (6,5) SWCNTs. The mixtures were exposed to UV light (Photochemical Reactors Ltd., Reading, UK; 400 W medium pressure mercury immersion lamp) for 20 min, then placed on a shaker for 1 hour at room temperature. Excess DNA was removed via dialysis against 1x DPBS with Millipore dialysis membranes (20 kDa MWCO). Solutions were removed from the dialysis devices and the volumes were normalized to 50 µL with 1x DPBS. This process was also carried out for controls in the absence of DNA.

### 2.4. Assembly of SWCNT–GFP Hybrids

SWCNT–GFP hybrids were assembled by mixing the DNA functionalized SWCNTs with varying amounts of GFP^DNA^ to optimize the extent of functionalization of SWCNTs and to minimize the amount of free GFP in solution. For example, in a typical assembly, the SWCNT–DNA solution from the previous step was mixed with GFP^DNA^ (5 nM final concentration) in DPBS. The solutions were incubated at room temperature overnight and were directly characterized by fluorescence and UV–Vis spectroscopy. The attached oligonucleotides on the SWCNTs and GFP^204azF^ were complementary to each other, with the sequences shown in Table 1.

### 2.5. Atomic Force Microscopy

SWCNT samples were deposited on freshly cleaved muscovite mica. First, the mica was treated with a solution of MgSO_4_ (0.5 M) and blown dry with argon. After formation, SWCNT–DNA–GFP hybrids were directly deposited on the mica. Solutions of SWCNT–DNA–GFP conjugates (5 µL) were cast on the substrate and allowed to incubate on a shaker for 10 min. Hybrids were imaged on a Bruker Dimension Icon atomic force microscope in PeakForce QNM mode, using ScanAsyst Fluid probes. Tris-acetate-EDTA (TAE) buffer pH 7 (Thermo Scientific, Loughborough, UK) with MgCl_2_ (50 mM; Sigma Aldrich) was used as an imaging buffer. Images were typically captured with 512 lines with a scan rate of 0.3 to 0.5 Hz. Samples were allowed to equilibrate on the AFM for 10 min before imaging took place to reduce the drift.

AFM images were analyzed using Nanoscope Analysis software. In general, images were flattened, and the Z-scale was normalized across all images. Structure heights were analyzed using the cut tool, from which height profiles were obtained. Following analysis, images were processed using Image J.

### 2.6. Fluorescence and UV–Vis Spectroscopy

Fluorescence spectroscopy was carried out on a Cary Eclipse fluorometer using a Hellma ultra micro quartz fluorescence cuvette. The samples were excited at 485 nm and the spectra were recorded from 500 nm to 600 nm. The emission and excitation slit widths were 10 nm and the scan rate was 120 nm/min. The PMT detector was set to 800 V.

UV–Vis spectroscopy was carried out using a Shimadzu UV-3600i Plus UV–VIS–NIR Spectrophotometer from 1300 nm to 400 nm. The measurement was collected with a single beam, and DPBS was used as a baseline.

## 3. Results

### 3.1. Purification of SWCNTs by Chirality

**Figure 1:**
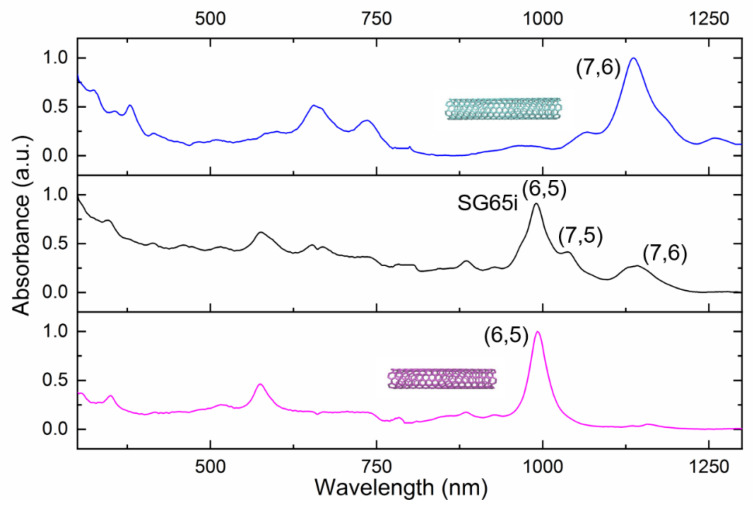
Normalized UV–Vis spectra of each SWCNT sample used.

DNA-wrapped SWCNTs were enriched in (6,5) and (7,6) using the method described by Lyu et al. (see also the Methods, Section 2.1) [25]. Chirality enrichment was confirmed via UV–Vis spectroscopy, where (6,5) and (7,6) variants were identified by their E_11_ absorbances at 993 nm and 1157 nm, respectively (Figure 1). Moreover, the enriched solutions of SWCNTs are presented as purple, indicative of (6,5), and blue, indicative of (7,6) (see Figure S1).

Based on the absorbance spectrum, the (6,5) species was found to be almost completely pure, with no E_11_ peaks from (7,6) and (7,5), both of which are prominent in the unsorted SG65i SWCNTs. SG65i SWCNTs contain approximately 40% (6,5). For the (7,6) batch, the absorbance peak at 1068 nm and the shoulder at 1185 nm suggest the presence of small amounts of (7,5) and (8,6) SWCNT variants. Importantly, no traces of (6,5) SWCNTs can be seen in this fraction, which allows us to investigate the assembly of SWCNT–GFP hybrids in the absence of (6,5) nanotubes.

### 3.2. Production of Azide–GFP and Functionalization of GFP with DNA

The GFP variant containing the non-canonical amino acid *p*-azido-L-phenylalanine (AzF) in place of residue Gln204 (Figure S2) was generated as described previously using an expanded genetic code approach [36,37,38]. The variant, termed GFP^204AzF^, was used because the AzF (and thus the linking site) lies close to the chromophore of GFP and has previously been shown to retain function on the attachment to SWCNTs and DNA [7,11,38]. To functionalize GFP with DNA, amine-modified DNA was reacted with BCN–NHS ester (see Figure S3 for details). The BCN–DNA was then reacted overnight with AzF–GFP, resulting in a GFP^DNA^ conjugate (Figure S4). The GFP^DNA^ conjugate was characterized and purified by PAGE. After extraction from the gel, the concentration of the GFP^DNA^ was determined to be 2.1 µM by UV–Vis (Figure S5) [37].

### 3.3. Formation of SWCNT–GFP Hybrids

To form the SWCNT–GFP hybrids, the DNA-wrapped SWCNTs were first functionalized with azido-DNA using a method which we have recently employed [9]. However, instead of using methanol as a solvent, the SWCNTs and DNA were reacted in the biologically relevant Dulbecco’s phosphate-buffered saline (DPBS). This was primarily to avoid precipitation of the SWCNTs that occurs in methanol and to conserve concentration across the various samples. Prior to functionalization with DNA, the concentrations of (6,5), (7,6), and mixed/unsorted SWCNTs (SG65i) were normalized by their E_11_ absorbances. The SG65i SWCNTs contained approximately 40% (6,5) SWCNTs; therefore, this solution was diluted to have 40% absorbance compared to the enriched (6,5) solution.

The reaction was initiated by irradiating the solution with UV light; this caused the formation of reactive nitrene groups, which subsequently reacted with the SWCNTs in a cycloaddition reaction (Figure S6). Although the reaction should predominantly target the exposed ends of the SWCNTs, there is the possibility of the nitrenes reacting along the sidewall of the SWCNTs due to potential gaps in the DNA coverage of the nanotube during the wrapping process. The efficiency of DNA attachment via this method was found to be quite high (~90%) in our previous study [9]. In the final step, GFP^DNA^ was combined with the SWCNT–DNA solutions to induce the assembly of the GFP–SWCNT hybrid via DNA hybridization.

The DNA sequences were designed to have a high melting temperature (T_M_) and minimal secondary interactions while ensuring the GFP was as close to the SWCNT as possible. Therefore, a length of 20 nucleotides was chosen and the sequence was designed to have a high GC content (60%). The complementary DNA sequences had a ∆G (free energy of each sequence binding to its complement)—as estimated by IDT DNA’s OligoAnalyzer—of −38.3 kcal/mole, giving a T_M_ of approximately 59 °C at 5 nM of GFP^DNA^.

**Figure 2:**
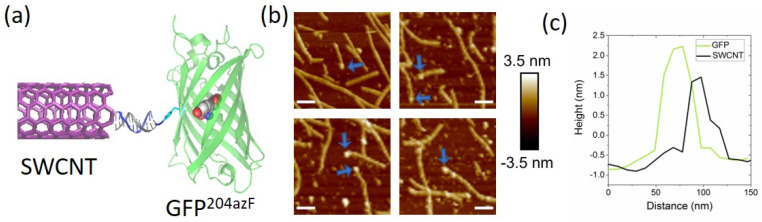
DNA-directed assembly of GFP and SWCNTs. (**a**) Schematic of SWCNT–GFP hybrid linked via DNA. (**b**) Representative AFM images carried out in fluid mode: proteins attached to SWCNTs are indicated with blue arrows. Scale bar = 100 nm, Z-scale = 7 nm. (**c**) Representative AFM height profiles of a GFP and an SWCNT within an assembled structure.

The formation of SWCNT–GFP hybrids was characterized using fluid AFM to avoid dehydration of the GFP which could lead to less accurate height measurements. Figure 2 shows representative topographical liquid-AFM images where the proteins are tethered predominantly to the terminal ends of individual SWCNTs (additional images can be found in Figure S7). This is due to the DNA-wrapping of the SWCNTs, which passivates the sidewalls of the tubes while leaving the ends of the SWCNTs exposed. This is in agreement with previous findings [7,8,9,39], although the reaction is not 100% selective, with marginal attachment also occurring on the sidewalls (see Figure S8). Only in the presence of complementary DNA has the hybridization-driven tethering of nano-moieties to the end of the CNTs been observed [8,9].

AFM height analysis of the hybrid structures confirmed that GFP was indeed tethered to the SWCNTs, as seen Figure 2b,c. GFP was found to have a height of approximately 2.5 nm, in agreement with previous work [40]. This could be distinguished from unfunctionalized SWCNTs which typically exhibited a height of 1.5 nm: AFM images of individual GFP^DNA^ conjugates and unfunctionalized SWCNTs can be seen in Figure S9. Based on our fluid AFM measurements, ~15% of SWCNTs exhibited GFP attachment. We believe the yield may be higher in solution, because tip-sample interactions in fluid AFM can cause removal of the GFP from the SWCNTs.

### 3.4. Optical Characterization of SWCNT–GFP Hybrids in Solution

**Figure 3:**
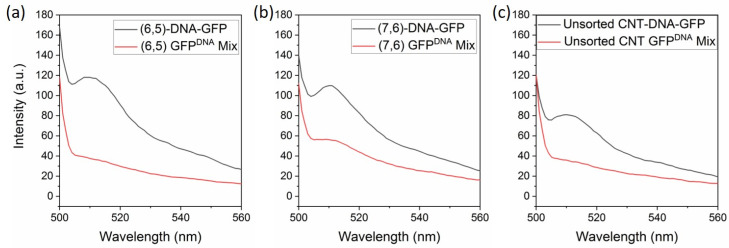
Fluorescence spectra of (**a**) enriched (6,5), (**b**) enriched (7,6), and (**c**) unsorted SG65i SWCNT–GFP hybrids (black) excited at 485 nm with a control mixture of each SWCNT variant with GFP^DNA^ (red).

To assess the coupling of SWCNTs and GFP, fluorescence spectroscopy was carried out (Figure 3). For each SWCNT form, the GFP-derived fluorescence (excitation at 485 nm) was measured. GFP^DNA^, when measured alone, did not exhibit detectable fluorescence (Figure S10) due the low concentration of GFP (2 nM). When GFP^DNA^ was mixed with unfunctionalized SWCNTs in solution, a moderate increase in fluorescence was observed, suggesting that electronic coupling was occurring between the two moieties. This is likely due to a protein corona, where proteins may electrostatically interact with the SWCNTs, leading to changes in the optical properties in SWCNT/GFPDNA mixtures [41,42,43]. When GFPDNA was directly coupled to SWCNTs via complementary DNA, a significant, and much larger, increase in emission intensity of the GFP occurred, suggesting a stronger electronic coupling between the SWCNT and GFP. Moreover, an increase in fluorescence intensity of the protein was observed for all SWCNT types coupled to GFP^DNA^.

When comparing the SWCNT–GFP hybrids’ emission with that of their control mixture, the (6,5)-GFP conjugate emission intensity was 3.1 times greater than that of its control. The DNA-linked conjugate of the (7,6) variant was 1.9 times higher than for the mix of GFP with SWCNT, whereas for the unsorted SG65i conjugate the observed emission of the GFP was 2.2 times higher than that of its control. These results suggest the occurrence of electronic coupling between GFP and SWCNTs, irrespective of which chirality is used. When the proteins and nanotubes were linked together via DNA to form a direct SWCNT–GFP hybrid, we observed a significant degree of coupling, as evidenced by the increase in fluorescence of the GFP.

As an additional control, the GFP^DNA^ conjugate was hybridized to an excess amount (10 equivalents) of the complementary sequence to assess if duplexed DNA contributed to the increase in fluorescence intensity. Notably, a slight decrease in intensity was observed in the presence of the complementary DNA, confirming that the increase in fluorescence intensity in the SWCNT–GFP hybrids was indeed attributable to the formation of the bioorganic nanohybrid (see Figure S11).

## 4. Discussion

Here, we have demonstrated the addressable assembly of DNA-linked SWCNT–GFP hybrids, employing enriched (6,5) and (7,6) SWCNT species and unsorted SG65i SWCNTs. The formation of the biohybrids resulted in electronic coupling between the GFP and all chiralities of SWCNT, as evidenced by an increase in fluorescence intensity of the GFP. The electronic coupling seen in the biohybrids occurred irrespective of which SWCNT solution was used.

In our previous investigations on SWCNT–GFP coupling, a decrease in fluorescence intensity was observed when GFP was conjugated to unsorted SWCNTs, both to the ends of the nanotubes via a linker and via direct attachment to the sidewalls [7,11]. Moreover, the bleaching times for GFPs directly attached to SWCNT sidewalls increased [11]. These previous results suggested the occurrence of electronic coupling between SWCNT and GFP, leading to a quenching of GFP’s emission. In contrast, this study showed that an increase in fluorescence intensity is observed for GFP attachment to SWCNTs via DNA, indicating the possibility of a directional coupling between the GFP and SWCNT with DNA potentially playing a role (e.g., through its intrinsic base pi-stacked and H-bond network present in double-stranded DNA). Further investigations are warranted to cast light on the mechanism involved in the electronic coupling observed here, on the effects of the protein on the electronic structure of the SWCNTs, and on the effect of GFP on the SWCNT emission, because protein attachment to SWCNTs can affect an increase or decrease in fluorescence intensity of SWCNTs [44,45].

The work described herein lays the groundwork for the assembly of SWCNT–protein structures controlled by DNA linkers, toward optical and electronic applications. The method of assembly can be expanded to other proteins of interest and may be useful in studying protein–protein interactions.

## Figures and Tables

**Table 1:** Oligonucleotide sequences used for the functionalization of GFP^204azF^ and SWCNTs.

Oligo Name	Sequence
(1)	5′-Amine-CCTGAGCCTGTAGTTGACCG-3′
(2)	5′-Azide-CGGTCAACTACAGGCTCAGG-3′

## Data Availability

The data presented in this study are openly available in Figshare at 10.6084/m9.figshare.14865795.

## Supplementary Materials

The following are available online at https://www.mdpi.com/article/10.3390/biom11070955/s1, Figure S1: Image of SWCNT chiralities, Figure S2: Representation of GFP^204AzF^, Figure S3: Scheme for BCN–DNA, Figure S4: Scheme for GFP^DNA^, Figure S5: UV–Vis of GFP^DNA^, Figure S6: Scheme for SWCNT functionalization, Figure S7: Additional AFM of SWCNT–GFP, Figure S8: AFM of sidewall GFP attachment, Figure S9: AFM images of uncoupled SWCNTs and GFP, Figure S10: Fluorescence spectra of GFP^DNA^, Figure S11: Fluorescence of GFP^DNA^ with complement.
