# Supplementary material for: DNA-Directed Assembly of Carbon Nanotube–Protein Hybrids
Source: Biomolecules. 2021 Jun 29;11(7):955. doi: 10.3390/biom11070955 (PMC8301810; doi:10.3390/biom11070955)
Supplement: Supplementary file 1 [file biomolecules-11-00955-s001.zip › biomolecules-1240209-supplementary.pdf]

DNA-directed assembly of Carbon Nanotube-Protein hybrids

Mark Freeley,[1] Rebecca E.A. Gwyther,[2] D. Dafydd Jones,[2]* and Matteo Palma.[1]*

1 Department of Chemistry, Queen Mary University of London, , London, E1 4NS, United Kingdom;

2 Molecular Biosciences Division, School of Biosciences, Cardiff University, Cardiff, CF10 3AX, Wales, United Kingdom;

Supplementary Information

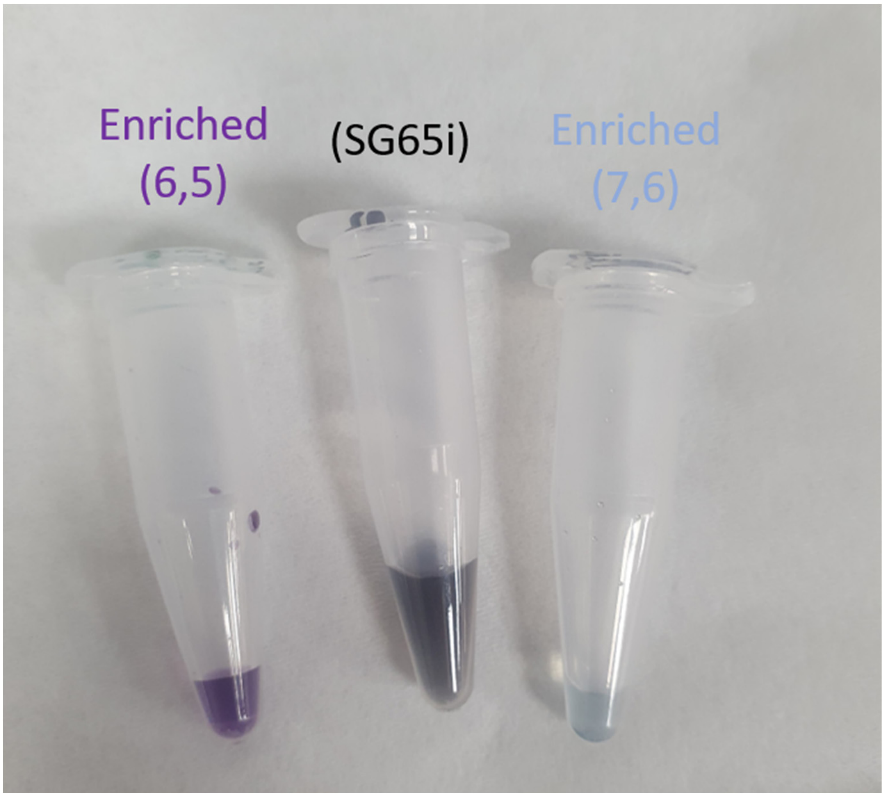

Figure S1. Image of the SWCNT species used.

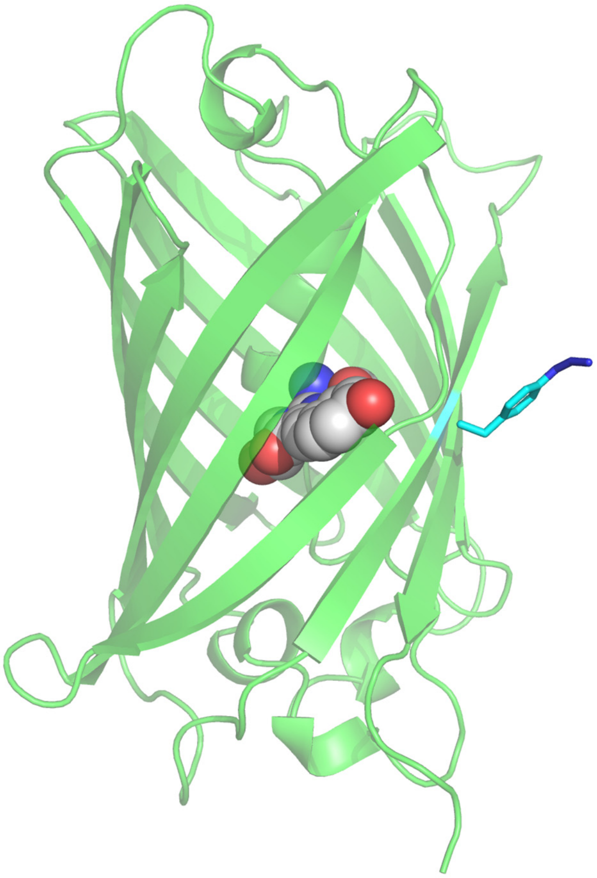

Figure S2. Representation of GFP^{204AzF}. The chromophore is shown as spheres and 204AzF is shown in sticks and coloured cyan.

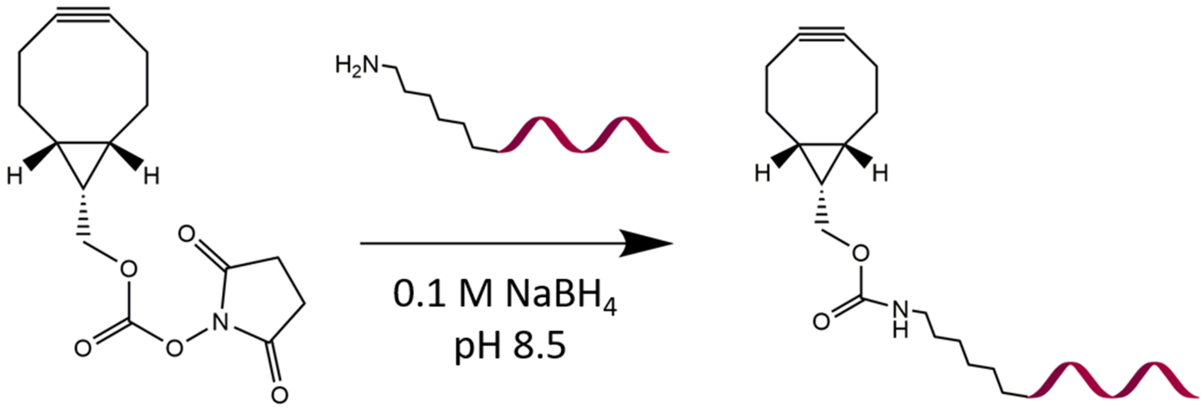

Figure S3. Scheme for the functionalisation of amine-modified oligonucleotide with BCN-NHS Ester.

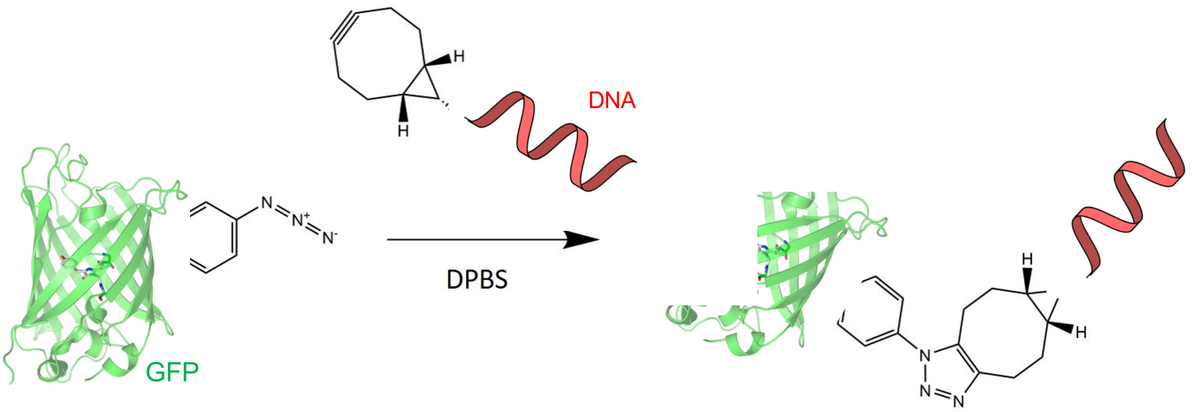

Figure S4. Scheme for the conjugation of BCN-DNA to azido-GFP.

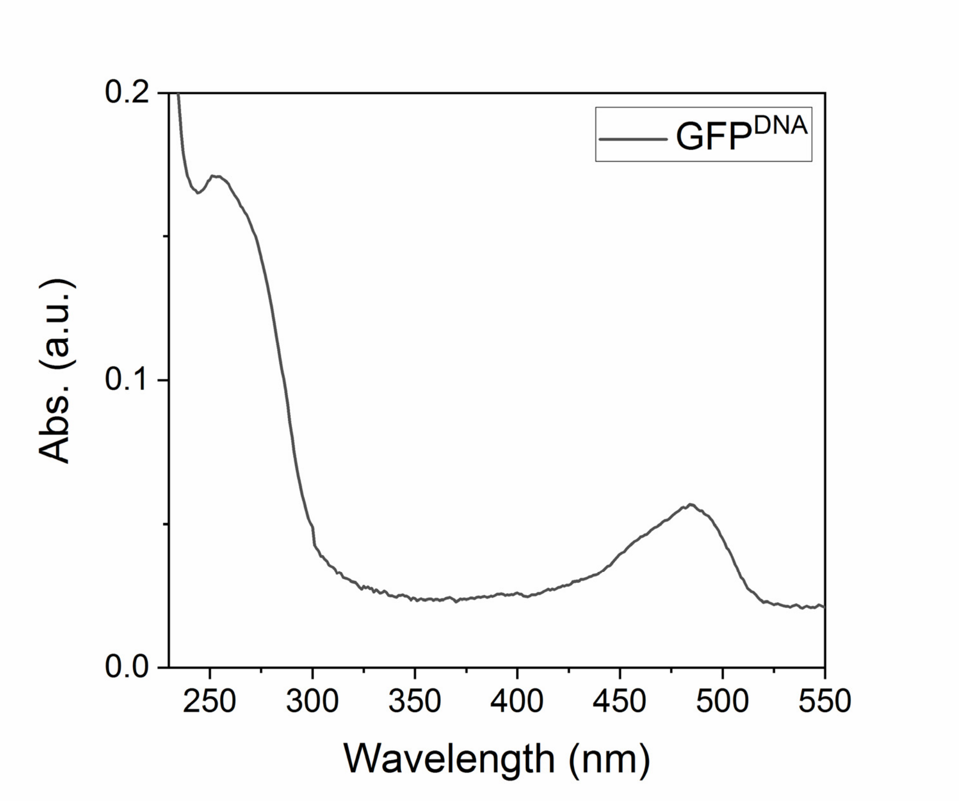

Figure S5. UV-vis spectrum of GFP-DNA conjugate.

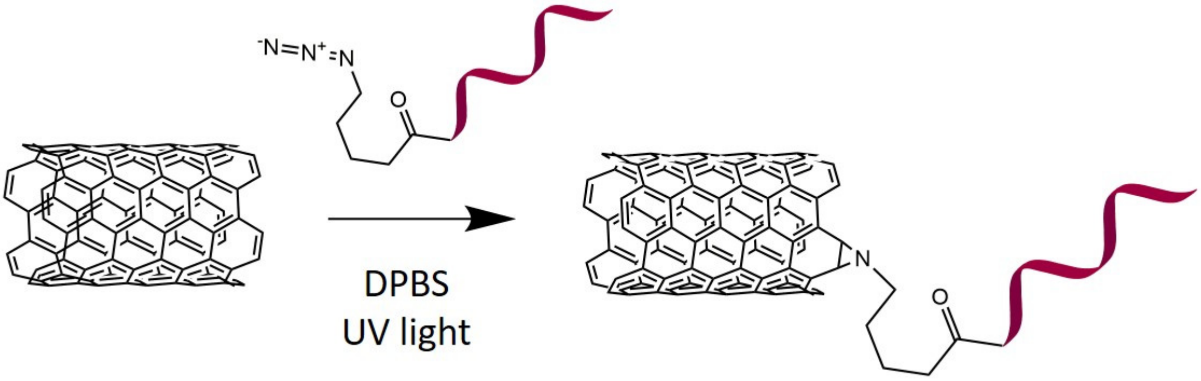

Figure S6. Scheme for the functionalisation of CNTs with azide-DNA via a UV-initiated reaction.

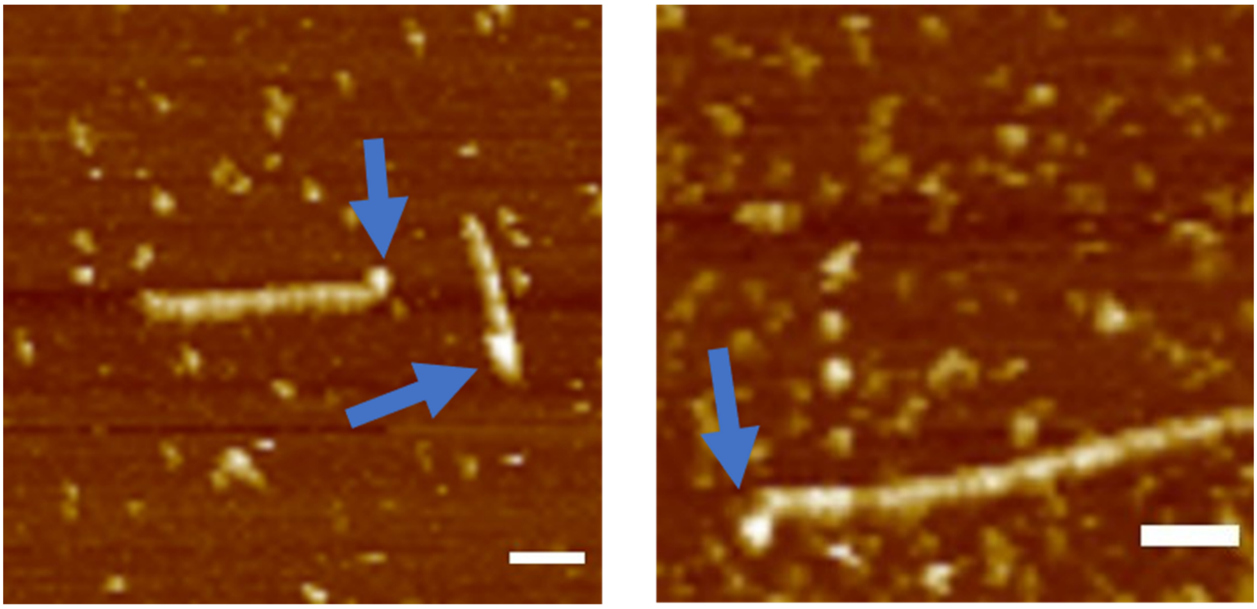

Figure S7. Additional AFM images of SWCNT-protein conjugates. Scale bar = 100 nm

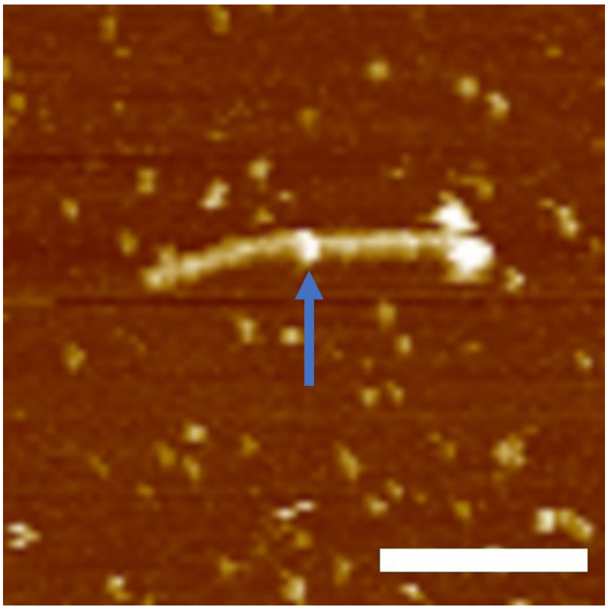

Figure S8. AFM image of protein attached to sidewall of SWCNT. Scale bar = 100 nm.

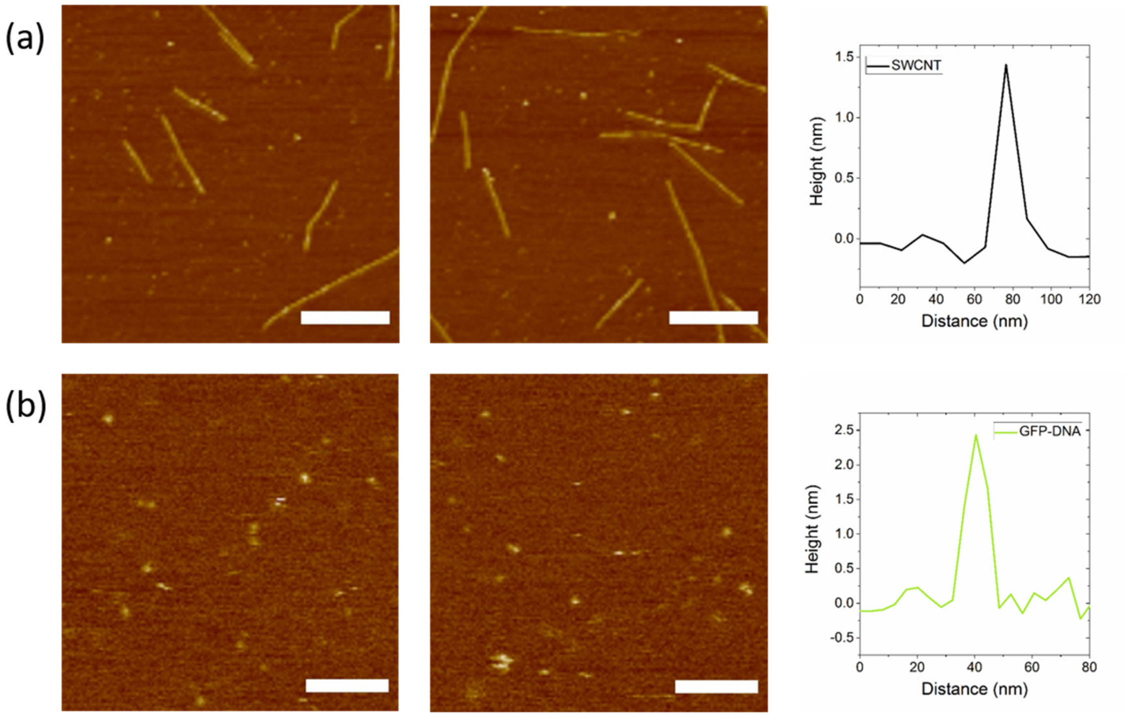

Figure S9. Fluid-mode AFM images of (a) unfunctionalized SWCNTs (scale bar = 250 nm) and (b) GFP-DNA (scale bar = 100 nm) with representative height profiles of each.

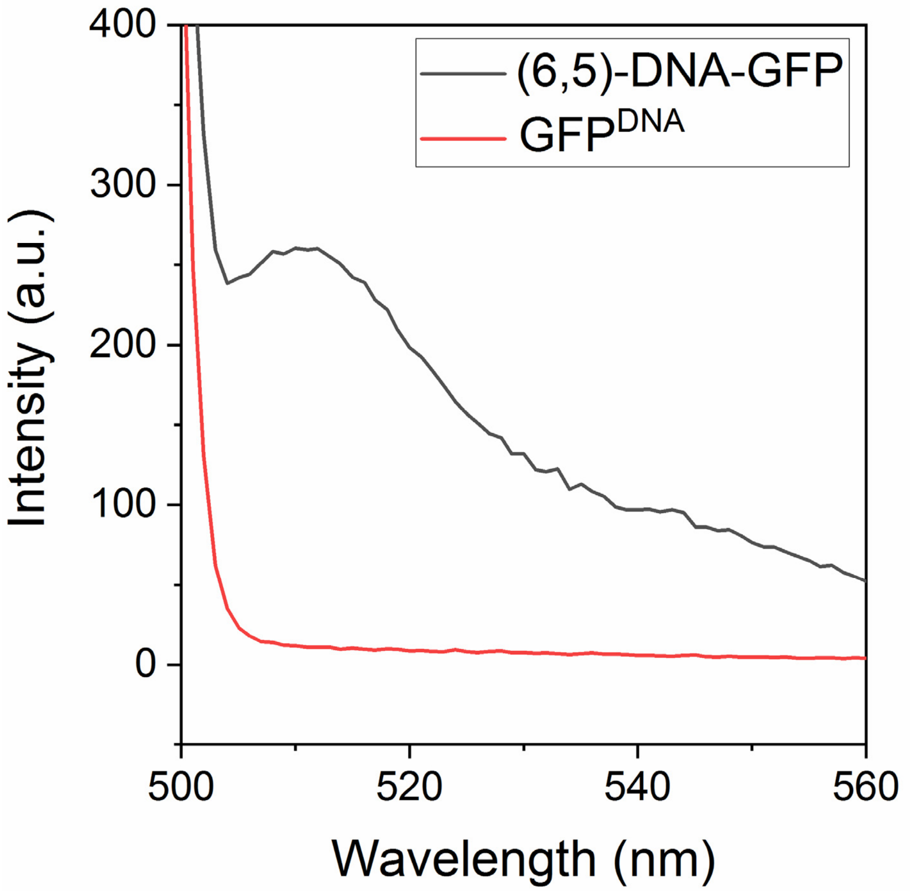

Figure S10. Comparison of the in-solution emission of SWCNT-GFP hybrids assembled with the DNA-functionalized (6,5) variant (black line) against GFP alone (red line), as measured by fluorescence spectroscopy.

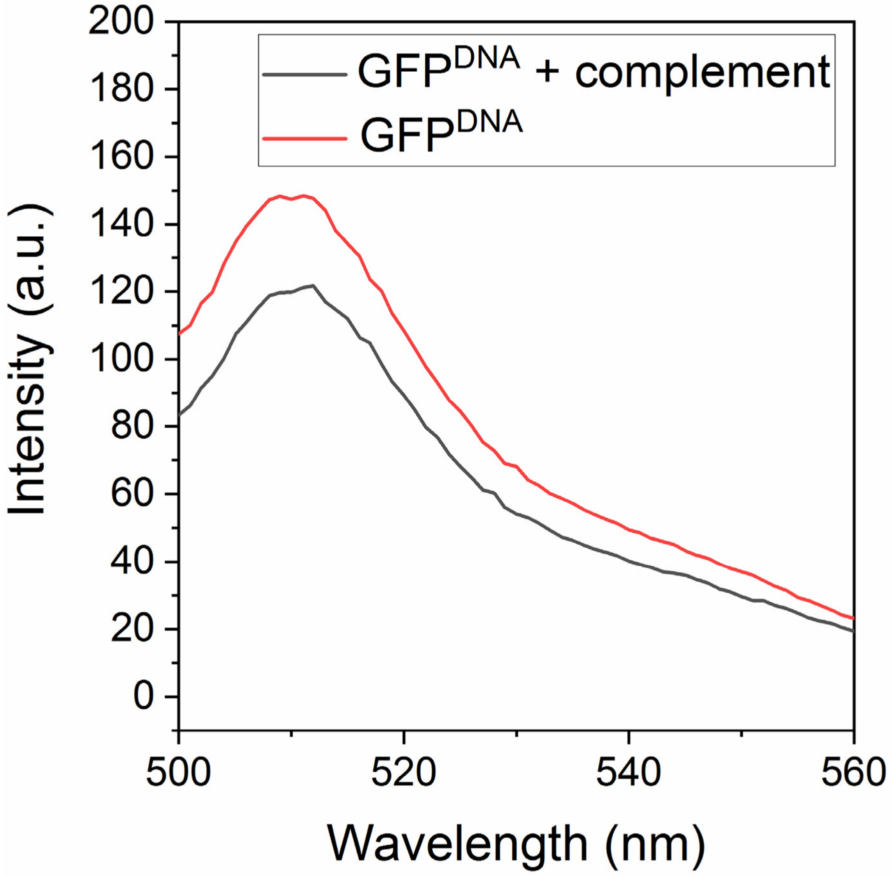

Figure S11. Fluorescence spectrum of GFP-DNA alone (50 nM) and with complementary DNA (500 nM).
